# The incidence of cutaneous squamous cell carcinoma in patients receiving voriconazole therapy for chronic pulmonary aspergillosis

**DOI:** 10.1007/s00210-020-01950-x

**Published:** 2020-08-21

**Authors:** Chris Kosmidis, Anna Mackenzie, Chris Harris, Rola Hashad, Fiona Lynch, David W. Denning

**Affiliations:** 1 Division of Infection, Immunity and Respiratory Medicine, School of Biological Sciences, Faculty of Biology, Medicine and Health, University of Manchester, Manchester Academic Health Science Centre, Manchester, UK; 2 National Aspergillosis Centre, Wythenshawe Hospital, Manchester University NHS Foundation Trust, 2nd Floor Education and Research Centre, Southmoor Road, Manchester, M23 9LT UK; 3 Department of Medical Microbiology and immunology, Faculty of Medicine, Alexandria University, Alexandria, Egypt

**Keywords:** Voriconazole, Squamous cell carcinoma, Skin cancer, Chronic pulmonary aspergillosis

## Abstract

Voriconazole has been associated with cutaneous squamous cell carcinoma (cSCC) in transplant patients but less is known about the risk in less severely immunosuppressed patients. Our aim was to estimate the incidence of cSCC after voriconazole exposure in patients with chronic pulmonary aspergillosis on a background of chronic lung disease. The notes of patients seen at a tertiary referral centre from 2009 to 2019 with chronic pulmonary aspergillosis were reviewed for the diagnosis of cSCC and voriconazole use documented. Among 1111 patients, 668 (60.1%) received voriconazole for longer than 28 days. Twelve patients received a diagnosis of cSCC; nine had used voriconazole. Mean duration of voriconazole use was 36.7 months. The crude incidence rate was 4.88 in 1000 person/years in those who had voriconazole and 2.79 in 1000 patient/years in those who did not receive voriconazole for longer than 28 days. On Cox regression, age (HR 1.09, 95% CI 1.02–1.16, *p* = 0.01) and male gender (HR 3.97, 95% CI 0.84–18.90, *p* = 0.082) were associated with cSCC. Voriconazole use was associated with a slightly increased risk, which was not significant (HR 1.35, 95% CI 0.35–5.20, *p* = 0.659). Voriconazole use beyond 28 days did not lead to a significantly increased risk of cSCC in a large cohort of patients with chronic pulmonary aspergillosis.

## Introduction

The spectrum of disease caused by Aspergillus encompasses allergic, chronic, saprophytic and invasive disease, depending on the degree of host immunosuppression. Chronic pulmonary aspergillosis (CPA) affects immunocompetent or mildly immunosuppressed patients with chronic lung disease, such as COPD, bronchiectasis, prior pulmonary TB or sarcoidosis (Smith and Denning 2011). Voriconazole is one of the first-line agents in CPA and is often used long-term. Guidelines suggest at least six months of treatment; however, in practice it may be used for longer durations, often for years, as response may be delayed and relapses are common (Denning et al. 2016).

Long-term voriconazole treatment is associated with hepatotoxicity, neurotoxicity, photosensitivity and, importantly, a risk of skin cancer. Photosensitivity is common and there is a clear association between its emergence and subsequent development of skin cancer (Cowen et al. 2010). It is hypothesised that voriconazole N-oxide, the primary metabolite of voriconazole and a UVB biproduct, potentiates UVA-mediated oxidative DNA damage or inhibits DNA repair (Ona and Oh 2015). Photosensitivity tends to resolve after cessation of the drug; it is not known if this is true of the skin cancer risk.

The association between voriconazole and cutaneous malignancy, particularly cutaneous squamous cell carcinoma (cSCC), is well established in severely immunocompromised patients like haematopoietic stem cell transplant (HSCT) or lung transplant recipients, with case reports in patients with renal transplant or HIV (Brunel et al. 2008; Hamandi et al. 2018; Kolaitis et al. 2017; Vanacker et al. 2008; Wojenski et al. 2015). These patients are already at increased risk of non-melanoma skin cancer and voriconazole was shown to increase this risk. Therefore, long-term voriconazole use should be pursued only if the benefit outweighs the risk, and only if frequent skin inspection is possible.

Whereas extensive data are available for the risk in the severely immunocompromised patients, the incidence, risk factors and clinical presentation of cSCC in other patient groups treated with voriconazole is not well described. At the National Aspergillosis Centre (NAC), more than 120 patients with CPA are seen yearly and a substantial number will be treated with voriconazole as first-line or salvage therapy, often for longer than 6 months. This large cohort of patients can provide valuable insights into the risk of cSCC in a population other than transplant recipients. The aim of this study was to estimate the incidence of cSCC after voriconazole exposure in a population without severe immune compromise.

## Methods

All patients with the diagnosis of CPA referred to the NAC (2009–March 2019) were included retrospectively. The start of observation was taken as the day voriconazole was started for patients who received voriconazole, and the first attendance in clinic for patients who did not receive voriconazole. Information was obtained from the medical notes for all subsequent visits until March 2019 unless the patient was discharged, lost to follow-up or died, in which case the last observation point was the last clinic visit for patients who did not develop cSCC and the time of diagnosis of cSCC in those who did. Voriconazole exposure was documented as a binary variable; patients receiving voriconazole for less than 28 days were considered non-exposed. We used this cut-off based on the practice to prescribe voriconazole for 28 days initially (as patients have chronic rather than acute disease, and are seen in the outpatient setting). Dosing and frequency of administration were not collected. Age, sex, underlying diseases, photosensitivity, mean of the last five voriconazole levels, location of cSCC, treatment and outcome were recorded for all patients with cSCC. The crude incidence rate and age-specific incidence rate of cSCC in patients who received voriconazole were calculated. Cox regression was performed to compare incidence of cSCC; parameters included were voriconazole use, gender and age.

## Results

A total of 1199 patients with the diagnosis of CPA were identified. Fifty-five had only one attendance with no follow-up and were excluded. For 32 there was no clinical information. One patient had been diagnosed with cSCC after having received voriconazole before referral to NAC and was excluded from further analysis. Of the 1111 patients with follow-up, 668 received voriconazole for 28 days or longer, 120 received voriconazole for less than 28 days and 302 never received voriconazole. Mean age was 66.6 years (range 21–96). Female were 475 (42.8%). Mean follow-up duration was 29.3 months (IQR 35.0) for patients who did not have voriconazole and 33.1 months (IQR 42.0) for those who had voriconazole. There were 12 cases of cSCC; nine in patients who received more than 28 days of voriconazole prior to cancer diagnosis (Table 1). All patients were white. All nine patients developed photosensitivity prior to cSCC diagnosis. The mean duration of voriconazole therapy was 36.7 months and the mean time from the start of therapy to cancer diagnosis was 47.2 months. The mean age of patients with cSCC was 65.6 years (SD 12.1) and 10 (83.3%) were male. All were white.

**Table 1:** Characteristics of patients with cutaneous squamous cell carcinoma (cSCC)

Patient	Age at diagnosis, sex	Underlying condition	Duration of voriconazole therapy and mean levels (mg/L)^a^	Time from start of therapy to cSCC diagnosis	Presence of phototoxicity before lesions	Type of cSCC and location	Outcome
1	49, M	Sarcoidosis on prednisolone 5 mg	4 y 3 m	4 y 3 m	1 y into therapy	Moderately differentiated left forehead	Resected. No relapse, radiotherapy
			1.92				
2	69, M	Metastatic hepatocellular carcinoma on sorafenib Asthma	4 y 6 m	4 y	3 y 10 m into therapy	Moderately differentiated scalp	Resected with local flap. 2 new scalp SCCs 10 m later, resected. New lesions 20 m later, awaiting biopsy, died from unrelated cause
			4.19				
3	69, M	Sarcoidosis on prednisolone 5 mg/day	4 y 7 m (with 3 m interruption)	4 y 7 m	8 m into therapy	Scalp	Resected. No relapse
			1.74				
4	80, M	COPD	2 y 11 m	2 y 11 m	1 y into therapy	Right ear, cervical lymph node metastases	Resected. Required plastic surgery with reconstruction
			2.04				
5	81, M	Bronchiectasis	3y 4 m	3y 4 m	1 y into therapy	Left ear	Resected. 2 further SCCs of hand 4 y later, awaited resection, died from unrelated cause
			1.78				
6	67, M	COPD	4y 1 m	4y 1 m	1 y 1 m into therapy	Well-differentiated right ear	Resected. Recurrence 6 m later, resected. SCC on right temple 12 m later, resected
			0.93				
7	80, M	Non-specific interstitial pneumonia on prednisolone 12.5 mg and cyclosporine	7 m	7 m	At same time	Two lesions Unknown area	Resected
			1.57				
8	77, M	Previous TB Right upper lobectomy	3y 8 m	7 y 1 m	9 m into therapy	Unknown area	Resected
			2.59				
9	67, M	Lung squamous cell carcinoma treated with cisplatin/etoposide and radiotherapy	4 y 5 m	4 y 7 m	5 m into therapy	Cheek	Resected
			3.75				
10	85, M	COPD Bronchiectasis	16 days	6 y 7 m	None	Scalp	Resected. Second SCC 6 m later, resected. Third SCC 18 m later, resected
11	78, F	COPD Prednisolone 10 mg	6 days	4 y 11 m	None	Left calf	Resected, required skin graft
12	75, F	ABPA Bronchiectasis	12 days	6 y 1 m	None	Poorly differentiated right cheek	Resected

^a^Mean of last five measurements of voriconazole levels before cSCC diagnosis. Levels recorded only for patients for whom voriconazole use had a temporal association with cSCC development. *y*, years; *m*, months; *M*, male; *F*, female

**Fig. 1:**
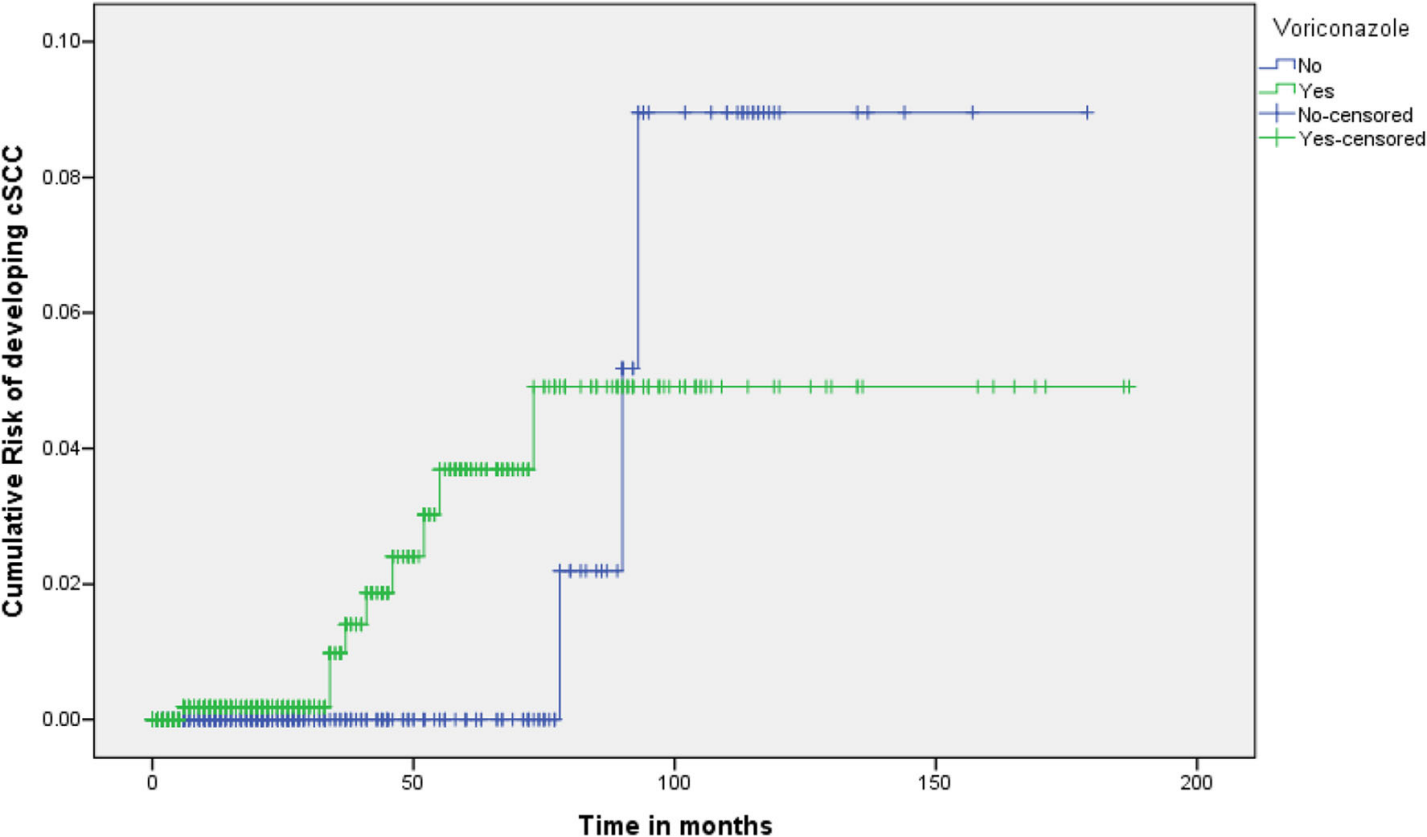
Kaplan-Meier curve of risk of developing cSCC (cutaneous squamous cell carcinoma) according to voriconazole status in patients with chronic pulmonary aspergillosis

The crude incidence rate was 4.88 per 1000 person/years in patients who received voriconazole for longer than 28 days and 2.79 per 1000 person/years in patients who did not receive voriconazole for longer than 28 days. Age-adjusted incidence rates on voriconazole were 13.3 per 1000 person/years in those aged > 74, 5.24 per 1000 person/years in those aged 65–74 and 2.33 per 1000 person/years on those younger than 65. On Cox regression, age (*p* = 0.01, HR 1.09, 95% CI 1.02–1.16) was significantly associated with risk of cSCC. Male gender (*p* = 0.082, HR 3.97, 95% CI 0.84–18.90) and voriconazole use (*p* = 0.659, HR 1.35, 95% CI 0.35–5.20) were associated with an increased risk of cSCC but did not reach statistical significance. A Kaplan-Meier curve of cSCC risk according to voriconazole status is presented in Fig. 1.

## Discussion

This is the first study, to our knowledge, to estimate the incidence of cSCC in a patient cohort on long-term voriconazole other than HSCT or solid organ transplant recipients. We found a slightly increased risk, although not significantly, of cSCC in patients with CPA treated with voriconazole. Patients with CPA usually have minimal or no immunosuppression; therefore, their risk of skin cancer should be comparable with that of the general population. The number of cases of cSCC observed in this cohort suggests that voriconazole may increase the risk of this cancer in patients without HSCT or solid organ transplant. Half of the patients who developed cSCC had some form of immune compromise, ranging from mild (e.g. 5 mg of prednisolone) to more extensive (e.g. previous chemotherapy for lung cancer). Therefore, it is possible that the risk of cSCC with voriconazole may be increased with any form of immunosuppression, not only the profound immunosuppression associated with transplant.

The increased cSCC rate we observed may not be entirely due to voriconazole, and could be attributed to our patients’ demographics, too, as CPA mainly affects middle-aged to elderly males, who have a higher incidence of cSCC (Brewster et al. 2007). All patients who developed cSCC after voriconazole use for longer than 28 days were male. cSCC is more common in males, and we observed a striking predominance of males in this series. In addition, chronic inflammation observed in CPA may be another factor predisposing to cancer. Although we did not compare voriconazole levels with those in patients who did not develop cSCC, the mean voriconazole levels were within recommended limits in patients who developed cSCC, suggesting that therapeutic level monitoring is not an effective way to prevent cancer development.

cSCC was diagnosed several years after voriconazole initiation in all but one patient, and in no patient was the diagnosis made within 6 months of treatment. This supports the recommendation that voriconazole use for up to 6 months is considered acceptable regarding the malignancy risk. Only one patient (case 8) developed cancer several years after voriconazole was stopped. This suggests that the risk of malignancy is reduced soon after voriconazole is stopped. For the three patients who were treated for only a few days (cases 10–12), cSCC developed several years later; therefore, the association with voriconazole is dubious. In addition, voriconazole-related cSCC described in case reports is multifocal and more aggressive (Vanacker et al. 2008). In our case series, three patients had relapses and one required reconstruction surgery due to extensive disease. Overall, we did not observe a particularly aggressive nature of disease.

This study has several limitations. The small number of cSCC cases limits the accuracy of our incidence estimation. We documented voriconazole use as a binary variable and did not correlate duration of treatment with cancer risk. We assumed a duration of less than 28 days as an arbitrary cut-off for long-term voriconazole exposure. This is similar to what was used by Hamandi et al. Using a different cut-off would not have significantly altered the results, as most patients who developed cSCC had either several months or only a few days of treatment (Table 1). In addition, we did not document daily or cumulative doses. The diagnosis of cSCC was done by retrospective review of notes, and therefore some information may have been missing. It is possible that some cases were not recorded by the clinician. Voriconazole use may have led to a more thorough assessment to rule out cancer compared with patients not on voriconazole. Finally, it was not possible to ascertain risk factors such as skin type or sun exposure, as these are not recorded routinely in notes.

In summary, in a cohort of patients with CPA, long-term voriconazole use was not associated with a significantly increased risk of cSCC to an extent previously seen in severely immunocompromised patients such as transplant recipients. However, the incidence was higher compared with non-exposed patients. In addition to counselling on the importance of using high factor sunscreen and avoiding sun exposure, regular monitoring for skin toxicity should be undertaken in all patients on voriconazole for longer than six months, particularly when photosensitivity is an issue.

## Data Availability

Not applicable.
